# Supplementary material for: Causal Relationship between Aging and Anorexia Nervosa: A White-Matter-Microstructure-Mediated Mendelian Randomization Analysis
Source: Biomedicines. 2024 Aug 16;12(8):1874. doi: 10.3390/biomedicines12081874 (PMC11351342; doi:10.3390/biomedicines12081874)
Supplement: Supplementary file 1 [file biomedicines-12-01874-s001.zip › Supplementary Figure/Figure S8/Leaveoneout plot of MR analysis for TL to ubm-b-2001.pdf]

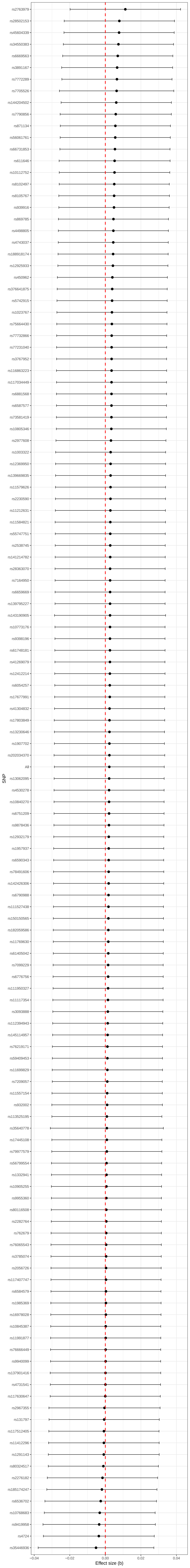
rs2763979
rs28502153
rs45604339
rs34550383
rs6669563
rs3891167
rs7772289
rs7705526
rs144204502
rs7790856
rs871134
rs56061761
rs66731853
rs611646
rs10112752
rs8102497
rs8105767
rs939916
rs869785
rs4498805
rs4743037
rs188918174
rs12925933
rs450962
rs376641875
rs5742915
rs1023767
rs75664430
rs77732866
rs77231040
rs3767952
rs116863223
rs117034449
rs6881568
rs6587577
rs73581419
rs10805346
rs2977608
rs1003322
rs12369950
rs139669835
rs11579626
rs2230590
rs11212631
rs11584821
rs55747751
rs2538745
rs141214782
rs28363070
rs7164950
rs6659669
rs139795227
rs143190905
rs10773176
rs9398196
rs61748181
rs41269079
rs12412214
rs6054257
rs17677991
rs41304832
rs17803849
rs13230646
rs1907702
rs202034370
All
rs13062095
rs4530278
rs10840270
rs6751209
rs9878436
rs12932179
rs1957937
rs6590343
rs78491606
rs142426306
rs6790988
rs111527438
rs150150565
rs182059586
rs11769630
rs61405042
rs7099229
rs6776756
rs111950327
rs11117354
rs3093888
rs112394943
rs145114957
rs76219171
rs59409453
rs11699829
rs7209057
rs11557154
rs932002
rs113525195
rs35640778
rs17445108
rs79977579
rs56799554
rs1332941
rs10905255
rs9955360
rs80116508
rs2282764
rs762679
rs76065543
rs3785074
rs2056726
rs117407747
rs6584579
rs1985369
rs16978028
rs10845387
rs11991877
rs76666449
rs9940099
rs137901416
rs4731541
rs117630647
rs2967355
rs131797
rs117512405
rs11412296
rs1291143
rs80324517
rs2276182
rs185174247
rs6536702
rs10768683
rs9419958
rs4724
rs35446936
SNP
-0.04
-0.02
0.00
0.02
0.04
Effect size (b)
